# A new paradigm for muscle contraction

**DOI:** 10.3389/fphys.2015.00174

**Published:** 2015-06-10

**Authors:** Walter Herzog, Krysta Powers, Kaleena Johnston, Mike Duvall

**Affiliations:** Faculty of Kinesiology, Engineering, Medicine and Veterinary Medicine, University of CalgaryCalgary, AB, Canada

**Keywords:** titin, actin, myosin, crossbridge theory, muscle contraction, eccentric, muscle stretching, force enhancement

## Abstract

For the past 60 years, muscle contraction had been thought to be governed exclusively by the contractile filaments, actin, and myosin. This thinking explained most observations for concentric and isometric, but not for eccentric muscle contractions. Just over a decade ago, we discovered that eccentric contractions were associated with a force that could not be assigned to actin and myosin, but was at least in part associated with the filamentous protein titin. Titin was found to bind calcium upon activation, thereby increasing its structural stability, and thus its stiffness and force. Furthermore, there is increasing evidence that the proximal part of titin binds to actin in an activation- and force-dependent manner, thereby shortening its free length, thus increasing its stiffness and force. Therefore, we propose that muscle contraction involves three filaments, actin, myosin and titin, and that titin regulates force by binding calcium and by shortening its spring length by binding to actin.

## Introduction and background

Muscle contraction has fascinated lay people and scientists for centuries. However, a good understanding of how muscle contraction occurs seemed only possible once microscopy techniques had evolved to a level where basic structural features, such as the regular cross striation patterns of fibers, could be observed in the late 19th century. In the early 20th century, a stimulated muscle was simply considered a new elastic body (Gasser and Hill, 1924). Shortening and work production took place with a fixed amount of energy that was stored in this body and evolved elastically through stimulation. However, this notion was proven false when Wallace Fenn demonstrated that muscle produced an increasing amount of total energy when increasing its mechanical work output; an observation that was in contradiction with Hill's elastic body theory (Fenn, 1923, 1924). Specifically, Fenn, who worked in the laboratory of Hill and measured heat and work production in frog muscles, found that a muscle allowed to shorten liberated more energy than a muscle held isometrically or a muscle that was stretched. This has become known as the Fenn effect in muscle physiology.

Prior to the 1950s, muscle contraction and force production were thought to be caused by the folding of long protein chains visible in the middle of the sarcomere. This shortening had been thought to be caused by lactic acid, but this theory was refuted by experiments demonstrating that contractions could be obtained in the absence of lactic acid in muscles poisoned with iodoacetic acid (Lundsgaard, 1930). The role of lactic acid was then replaced briefly with phosphorylcreatine breakdown, until it was discovered that this reaction merely served to re-phosphorylate ADP into ATP. Thereafter, filament shortening became associated with the hydrolysis of ATP into ADP and inorganic phosphate.

In the early 1950s, careful analysis of A-band dimensions revealed that myosin filaments were not substantially shortening under a variety of contractile conditions, and thus, could not account for muscle contraction, force production and the large length changes that muscle tissue can undergo (Huxley, 1953). In two seminal papers, arrived at independently, Hugh Huxley and Andrew Huxley proposed that muscle contraction occurred not by shortening of the myosin filaments, but by the relative sliding of two sets of filaments, actin, and myosin (Huxley and Hanson, 1954; Huxley and Niedergerke, 1954). In 1957, Andrew Huxley proposed how this relative sliding might occur, and provided a mathematical framework for what is now known as the cross-bridge theory of muscle contraction (Huxley, 1957). This paper, which has been cited over 3000 times (Google Scholar, June 2014), outlines in broad strokes how we think about muscle contraction today. The success of this paper is insofar surprising as Huxley never intended to publish it, and thought of the mathematical formulation of the cross-bridge theory merely as a preliminary idea (Huxley, personal communication, August 1999). He sent the paper to his friend and editor of *Biophysics and biophysical Chemistry*, who, to Huxley's surprise, suggested publishing it.

## Unaccounted observations

The initial two state model of the cross-bridge theory published in 1957 underwent several reformulations, although the basic premise remained unchanged. Briefly, in the cross-bridge model, contraction and force production is achieved by extensions (cross-bridges) from the thick (myosin) filaments that interact cyclically with the thin (actin) filaments and exert force between these two sets of filaments to produce shortening. Each cycle of attachment and detachment of a cross-bridge is associated with the hydrolysis of one molecule of ATP. Therefore, the regulation of force is governed exclusively by the contractile proteins actin and myosin, while structural proteins provide passive forces upon muscle elongation that are determined exclusively by their viscoelastic properties.

**Figure 1:**
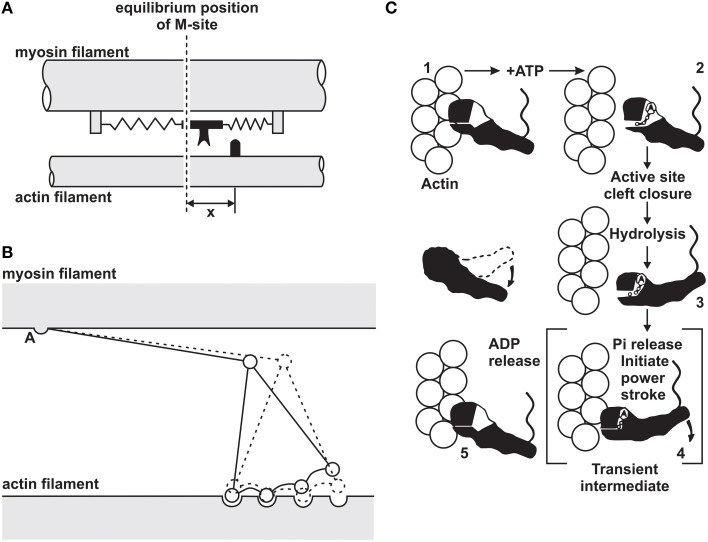
**Evolution of the cross-bridge model of muscle contraction**. **(A)** Original 2-state model proposed by Huxley (1957). **(B)** Multi-state cross-bridge model with rotating head as proposed initially by Huxley (1969) and mathematically described by Huxley and Simmons (1971). **(C)** Multi-state model based on the atomic structure of cross-bridges and actin attachment sites as proposed by Rayment et al. (1993).

In 1969, the cross-bridge theory was amended with the idea that cross-bridges produce force and shortening through rotation requiring multiple attached states (Huxley, 1969; Huxley and Simmons, 1971), and in 1993, a detailed description of the atomic structure of cross-bridges and the corresponding actin attachment sites revealed a cross-bridge stroke that included rotation of the cross-bridge around a fixed element of the cross-bridge head that attached uniquely to the actin attachment site (Figure 1) (Rayment et al., 1993).

In his original description of the cross-bridge theory, Huxley (1957) was able to predict forces for concentric contractions accurately. Specifically, Huxley derived a set of rate constants for the attachment/detachment kinetics of cross-bridges that accurately predicted “the best available data at the time,” the concentric force-velocity relationship of striated muscles (Hill, 1938). Furthermore, the cross-bridge theory also explained beautifully the isometric force as a function of fiber and sarcomere lengths (Gordon et al., 1966). However, the forces and energetics for eccentric contractions (actively stretched muscles), were not predicted accurately: they were much too big (Huxley, 1957). Also, the well-known results on residual force enhancement following active stretching of muscles (Abbott and Aubert, 1952; Edman et al., 1982) could not be predicted conceptually (Walcott and Herzog, 2008) or numerically (Herzog et al., 2012a,b; Herzog, 2014).

**Figure 2:**
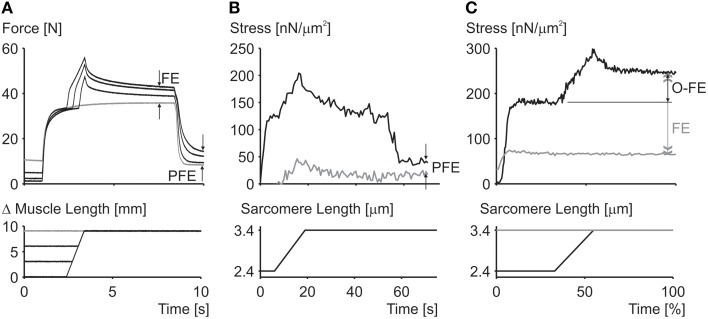
**Residual force enhancement in skeletal muscles**. **(A)** Residual force enhancement (FE) and passive force enhancement (PFE) in a whole muscle preparation (cat soleus at 37°C). **(B)** Passive force enhancement (PFE) in a single myofibril preparation (rabbit psoas at 21°C). **(C)** Force enhancement (FE) and force above the isometric plateau (O-FE) in a single sarcomere preparation (rabbit psoas at 21°C). The gray line in **(A)** represents the isometric reference contraction while the black lines represent active stretch contractions followed by an isometric contraction. The gray line in **(B)** represents passive force during myofibril stretching and the black line the corresponding active force; deactivation occurred at about 55 s. The gray line in **(C)** represents the isometric reference force while the black line represents the experimentally enhanced force following an active stretch.

Residual force enhancement is an acknowledged property of skeletal muscle (Edman et al., 1982). It describes the increase in steady-state isometric force following an active muscle stretch, compared to the corresponding purely isometric force at the same length and same activation (Figure 2). Residual force enhancement has been observed in whole muscle preparations, activated voluntarily (Oskouei and Herzog, 2005) or through electrical stimulation (Lee and Herzog, 2002), in single intact and skinned fibers (Edman et al., 1978, 1982; Sugi and Tsuchiya, 1988; Rassier et al., 2003c; Peterson et al., 2004; Lee and Herzog, 2008; Joumaa and Herzog, 2013), in myofibrils (Rassier et al., 2003a; Joumaa et al., 2008) and in single, mechanically isolated sarcomeres (Leonard et al., 2010) and half sarcomeres (Joumaa et al., 2008). Residual force enhancement cannot be predicted using the cross-bridge theory (Walcott and Herzog, 2008), because the rate constants of cross-bridge attachment/detachment do not depend on time but only on the relative location of the cross-bridge's equilibrium position relative to its nearest attachment site on actin (Huxley, 1957). Therefore, an explanation for residual force enhancement needed to be found that would not undermine the cross-bridge theory. Thus, for more than half a century, residual force enhancement was explained conceptually, albeit not numerically, with the idea of instability of sarcomeres on the descending limb of the force-length relationship (Hill, 1953) and the associated development of large sarcomere lengths non-uniformities (Morgan, 1990, 1994).

## Explanation of residual force enhancement (using sarcomere length non-uniformity)

**Figure 3:**
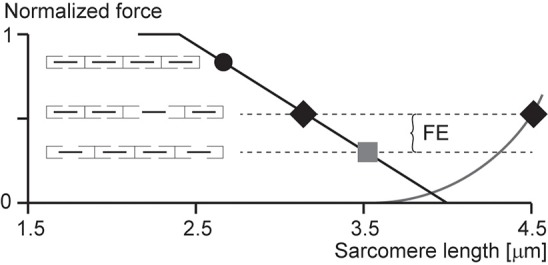
**Force enhancement (FE) based on the sarcomere length non-uniformity theory**. Isometric contractions of muscles on the descending limb of the force-length relationship are thought to occur with sarcomeres of uniform lengths (black circle and gray square). However, if a muscle is stretched from a short length (black circle) to a long length (gray square), some sarcomeres are thought to become overstretched (popped—right black diamond) while others are thought to be stretched only minimally (left black diamond), thus producing an average sarcomere length the same as that of the long isometric contraction (gray square), but producing more force because of the more favorable position of the short sarcomeres (left black diamond) and the passive forces of the overstretched sarcomeres (right black diamond).

According to the sarcomere length non-uniformity theory, sarcomeres on the descending limb of the force-length relationship are unstable (Hill, 1953; Allinger et al., 1996; Zahalak, 1997). This instability is thought to be caused by a “weakening” behavior of sarcomeres (negative stiffness). Therefore, for a perturbation, such as active stretching of muscle on the descending limb of the force-length relationship, sarcomeres were thought to be destabilized, causing a quick, uncontrolled over-stretching (popping) of some sarcomeres at the expense of others that only stretch slightly, not at all, or might even shorten by a small amount. The popped sarcomeres were thought to achieve force equilibrium with the short (active) sarcomeres through passive forces that become high at long lengths. Force enhancement was then explained with the idea that isometric contractions on the descending limb do not produce a sufficient perturbation to sarcomeres, thus sarcomeres remain relatively uniform and thus produce a force in accordance with actin-myosin filament overlap (Gordon et al., 1966). In contrast, a muscle that is stretched actively was thought to produce perturbations that result in instabilities and large sarcomere length non-uniformities that give rise to two distinct sets of sarcomere lengths. The steady-state force following active stretch was then thought to be greater than the purely isometric force because the active sarcomeres are shorter following active stretch compared to the purely isometric contraction (and thus can produce more force), and the passive sarcomeres are pulled to such lengths that their passive forces match the forces of the short, active sarcomeres (Figure 3).

In the following, we would like to identify predictions that are direct outcomes of the mathematical formulation of the sarcomere length non-uniformity theory. We will discuss these theoretical predictions in view of existing experimental evidence, and then will attempt to draw conclusions about the appropriateness of this theory.

## Predictions based on the sarcomere length non-uniformity theory

The theoretical models of the sarcomere length non-uniformity theory provide uniquely testable predictions (Zahalak, 1997; Campbell, 2009). Some of the primary predictions have been identified and discussed by proponents and opponents of this theory (Morgan et al., 2000; Herzog and Leonard, 2006, 2013; Herzog et al., 2006; Morgan and Proske, 2006; Edman, 2012). However, other equally obvious predictions, have received less attention, possibly because of the difficulties in testing them.

The primary predictions of the sarcomere length non-uniformity theory that have been discussed in previous works might be summarized as follows:

1. Sarcomeres are unstable on the descending limb of the force-length relationship following active stretching (Morgan, 1990, 1994).
2. Instability of sarcomere lengths, and thus the development of sarcomere length non-uniformities (and associated force enhancement) can only occur on the unstable descending limb but not the stable ascending limb of the force-length relationship (Allinger et al., 1996; Zahalak, 1997).
3. Forces in the force enhanced state cannot exceed the purely isometric forces at the plateau of the force-length relationship (Edman et al., 1982; Rassier et al., 2003c).
4. Force enhancement cannot occur in a single sarcomere preparation (Leonard et al., 2010).

Secondary predictions that follow directly from the mathematical framework and conceptual thinking of the sarcomere length non-uniformity theory that have received less, or no attention, may be summarized as follows:

5. Isometric contractions on the descending limb of the force-length relationship are associated with sarcomeres of essentially uniform length (Morgan, 1990, 1994).
6. Sarcomere length non-uniformities increase when a muscle is actively stretched compared to the corresponding purely isometric contractions (Morgan, 1990, 1994; Allinger et al., 1996).
7. Sarcomere lengths following active muscle stretching will have two distinct values (Allinger et al., 1996; Walcott and Herzog, 2008).

The primary predictions (1–4) of the sarcomere length non-uniformity theory have been discussed extensively (Herzog et al., 2006, 2012a,b; Edman, 2012; Herzog, 2014) but for completeness are summarized here briefly.

**Figure 4:**
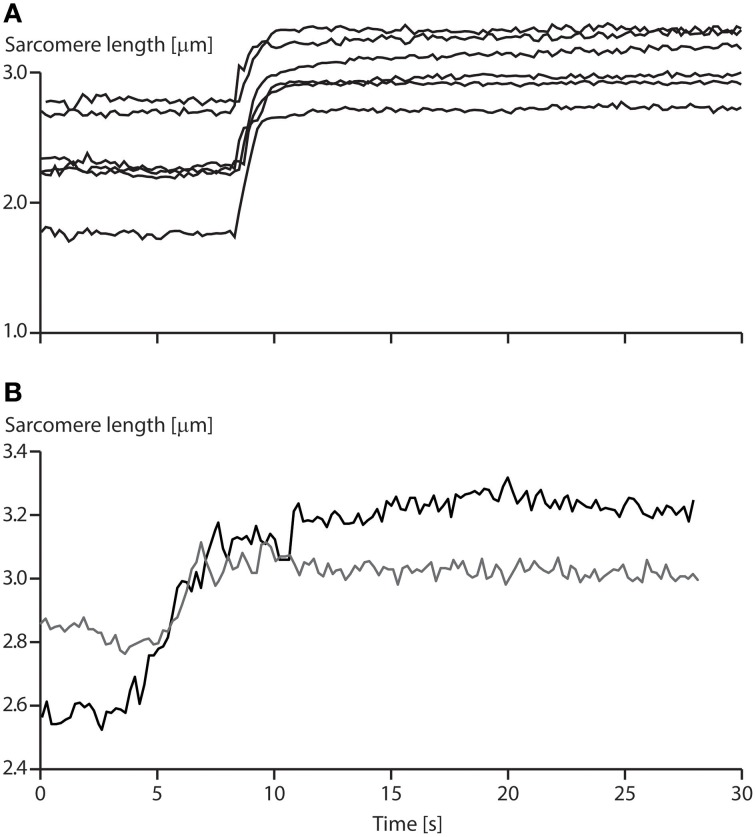
**Sarcomere length stability on the descending limb of the force-length relationship**. **(A)** Sarcomere lengths as a function of time for a six sarcomere myofibril stretched on the descending limb of the force-length relationship. Note that none of the sarcomeres is rapidly stretched beyond actin-myosin filament overlap (popped approximately 4.0 μm), but that they stay relatively constant in length for a 20 s period following active stretching. **(B)** Sarcomere lengths of two specific sarcomeres from a single myofibril. Note, when the myofibril is stretched, the initially short sarcomere becomes the long sarcomere and vice versa.

1. **Instability of sarcomeres on the descending limb of the force-length relationship:** More than 30 years ago, when first reading about sarcomere length instability on the descending limb of the force-length relationship, I asked myself the question: why would nature evolve a universal motor for contraction that was unstable and would tear itself apart, over more than half of its potential working range? After developing a setup for single myofibril testing, it was the first question I wanted to be answered. Stretching of serially arranged sarcomeres onto the descending limb of the force-length relationship did not produce sarcomere length instabilities (Rassier et al., 2003b); i.e., there was no quick, uncontrolled popping of the “weakest” sarcomeres, as predicted by the theory (Morgan, 1990, 1994). Rather, sarcomeres were perfectly stable at vastly differing lengths on the descending limb of the force-length relationship (Figure 4A), an observation that still needs satisfactory explanation. Frequently, sarcomeres that were shorter compared to other sarcomeres prior to active stretching, were longer after stretching (Figure 4B), a finding that is incompatible with the sarcomere length non-uniformity and cross-bridge theories.
2. **Force enhancement on the ascending limb of the force-length relationship:** The ascending limb of the force-length relationship has a positive slope, and thus strengthening character which produces inherent sarcomere length stability (Epstein and Herzog, 1998). Therefore, according to the sarcomere length non-uniformity theory, there should be no force enhancement on the ascending limb of the force-length relationship. However, in the very first systematic analysis of force enhancement, Abbott and Aubert (1952) reported force enhancement on the ascending limb of isolated muscle preparations. This initial finding was supported by further observations on whole muscles (Morgan et al., 2000), and single fibers (Peterson et al., 2004). However, force enhancement on the ascending limb tends to be small compared to the descending limb, and thus, although consistently observed, may not always be acknowledged (Morgan et al., 2000; Edman, 2012).
3. **Enhanced force above the isometric plateau forces:** In the sarcomere length non-uniformity theory, the steady-state isometric forces, independent of the history of contraction, cannot exceed the isometric forces obtained at the plateau of the force-length relationship. Since, the short sarcomeres in this theory are the active force producers, and since they must be within a region of actin-myosin filament overlap, the maximal force they can produce is that obtained at optimal sarcomere length represented by the plateau of the force-length relationship (Gordon et al., 1966). However, forces in the enhanced state exceeding those of purely isometric contractions at optimal lengths have been observed as early as 1978 (Edman et al., 1978), although these authors later revised their results. However, enhanced forces clearly exceeding the isometric plateau forces were found in a series of subsequent studies on whole muscles (Schachar et al., 2004), single fibers (Rassier et al., 2003c; Lee and Herzog, 2008), and most importantly, in single myofibrils and single, mechanically isolated sarcomeres (Leonard et al., 2010) (Figure 2). In conclusion, isometric steady-state forces following active muscle stretch can exceed the purely isometric forces at optimal length by a substantial amount.
4. **Force enhancement in a single sarcomere:** Obviously, if force enhancement requires the development of sarcomere length non-uniformities, as indicated in Figure 3, then force enhancement should never occur in a single sarcomere. The classic work by Tim Leonard was the first published research on force enhancement in a mechanically isolated single sarcomere preparation (Leonard et al., 2010). In that study, 10 single sarcomeres were isolated and stretched from optimal length (2.4 μm) to a length of 3.4 μm, and compared to the steady-state forces obtained for purely isometric contractions. Force enhancement was 190% on average and the enhanced forces exceeded the purely isometric forces at optimal length by an average of 37%. It has been suggested that these results might have been obtained due to the development of half-sarcomere length non-uniformities in this single sarcomere preparation. However, if so, the question remains how a single half-sarcomere can produce forces in excess of its isometric force at optimal length. Needless to say that one must be careful of results from a single study that have not been repeated in other laboratories, but for lack of evidence to the contrary, we accept that force enhancement is a sarcomeric property, and can occur in the absence of multiple sarcomeres of vastly different lengths.

The secondary predictions (5–7) of the sarcomere lengths non-uniformity theory have received much less, or no attention in the past, but seem equally relevant and will be discussed in the following.

5. **Uniform sarcomere lengths for isometric contractions:** For the sarcomere length non-uniformity theory to work, specifically for it to account for the residual force enhancement property of skeletal muscle, it is necessary that sarcomeres are essentially uniform for isometric contractions. Active stretching is then thought to be the stimulus that produces sarcomere length instability and associated length non-uniformities. There have been extensive reports that sarcomere lengths in muscle fibers are highly non-uniform (Huxley and Peachey, 1961), thus requiring specialized approaches when studying sarcomere force-length properties (Gordon et al., 1966). Sarcomere length non-uniformities have been primarily observed as average sarcomere length variations across single fibers, but more recently have been demonstrated for single sarcomeres in whole muscles (Llewellyn et al., 2008), and in single myofibrils (Figure 4). When quantifying sarcomere lengths in passive and active human muscles, Llewellyn et al. (2008) noticed variations in sarcomere lengths of 20% in a radius as small as 25 μm, while we found peak sarcomere length non-uniformities for purely isometric contractions of 37% in isolated myofibril preparations. Sarcomere lengths variations have been shown to range from 1.7 to 3.5 μm in frog semitendinosus fibers at rest (Huxley and Peachey, 1961). Taken together, these results suggest that sarcomere lengths non-uniformities are a natural occurrence of resting and activated muscle preparations at all structural levels. Thus, observing sarcomere length non-uniformities after stretch or shortening contractions should not imply that the dynamics of muscle contraction produced these non-uniformities, nor should these non-uniformities be thought to be the cause of specific mechanical properties of muscle without careful analyses.
6. **Increase in sarcomere length non-uniformity with active stretching of muscle:** One of the tenets of the sarcomere length non-uniformity theory in explaining force enhancement is the idea that sarcomere lengths become non-uniform during active stretching on the descending limb of the force length relationship, while they remain uniform with passive stretching and subsequent isometric contraction. If sarcomere lengths are indeed more non-uniform in the force enhanced compared to the isometric reference state has never been tested systematically. Initial work on this topic was done by stretching active single fibers or whole muscle preparations, and fixing them quickly following the stretch. These “stretched” preparations were then compared histologically to corresponding preparations that were activated isometrically or were allowed to actively shorten (Julian and Morgan, 1979; Morgan et al., 1982). These experiments typically showed an increased number of overstretched (popped) sarcomeres in the actively stretched muscles compared to those not undergoing active lengthening. These experiments have the advantage that they are performed in intact preparations, but have the disadvantage that overstretching of the sarcomeres cannot be accounted for by instability, rather than, for example, pre-existing structural damage of the muscle, and that only a tiny fraction of the whole muscle was analyzed for overstretched sarcomeres, thus making generalization difficult.

Reports of stability or sarcomere lengths non-uniformities after active stretching suggest that, if anything at all, sarcomeres are more stable (Edman et al., 1982), and sarcomere lengths are more uniform following active muscle stretching compared to the corresponding purely isometric reference contractions (Joumaa et al., 2008). Ongoing work in our lab has focused on measuring sarcomere length non-uniformities in isolated myofibril preparations in the force enhanced and normal isometric reference states. Preliminary results suggest that there is no systematic increase in sarcomere length non-uniformity, as quantified by the range and variance of sarcomere lengths following eccentric contractions on the descending limb of the force-length relationship compared to the corresponding purely isometric reference contractions.

7. **Two distinct sarcomere lengths following active muscle stretching:** In a muscle or fiber preparation, sarcomeres are not only connected serially but also in parallel. Therefore, force transmission by a sarcomere is not only determined by the force it exerts, but also by the forces acting on it in series and in parallel. However, in an idealized preparation, as typically modeled theoretically, sarcomeres are assumed to be perfectly in series. Such an idealization is achieved experimentally when using single myofibril preparations, which makes this preparation uniquely attractive to study sarcomeric properties. According to the sarcomere length non-uniformity theory, sarcomeres in series (in a myofibril) should be separated into two distinct groups with two distinct lengths when actively stretched. One group representing the sarcomeres that were not or only slightly stretched, the other representing the overstretched (popped) sarcomeres. Quantification of sarcomere lengths from a variety of published studies suggest that sarcomere lengths do not fall into two distinct lengths categories, but rather, are distributed over a range of lengths (e.g., Figure 4). Careful analysis of the sarcomere lengths of twelve myofibrils did not reveal a single one of them having two distinct sarcomere lengths in the force enhanced state thus defying this specific prediction.

## Further considerations

### Force-length relationship

**Figure 5:**
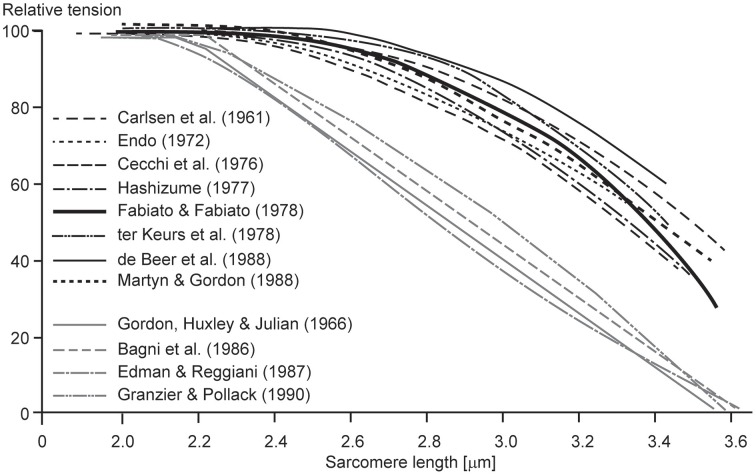
**Descending limb of the sarcomere force-length relationship**. Relative tension as a function of sarcomere lengths obtained from fixed end contractions (black lines) and from segment-clamped experiments (gray lines). Note that when the natural development of sarcomere length non-uniformities is prevented by segment clamping, the isometric forces are severely decreased (Pollack, 1990).

One of the most puzzling results of muscle physiology is the different shapes of the descending limbs of the force-length relationships for so-called “fixed-end” and “segment-clamped” conditions (Figure 5). In fixed-end experiments, the two ends of a fiber are fixed to a motor and a force transducer, respectively, and measurements of isometric force are made as a function of fiber lengths (Pollack, 1990). In segment-clamped experiments, a small mid-section of a fiber with relatively uniform sarcomeres is identified and marked, and its length is kept constant using length feedback by carefully adjusting the entire fiber length. Isometric forces are then expressed as a function of the sarcomere lengths in the fixed section of the fiber. For the fixed-end contractions, sarcomeres are allowed to take on their “normal” non-uniform length distribution (Pollack, 1990), while in the segment-clamped approach, sarcomere lengths are kept constant for the clamped segment (Gordon et al., 1966). Isometric forces on the descending limb of the force-length relationship in the fixed-end contractions have been reported to be substantially greater than those obtained in the corresponding segment-clamped experiments. Therefore, it appears that allowing sarcomeres to attain their natural non-uniform length distribution during purely isometric contraction enhances force, while attempting to keep sarcomere lengths artificially uniform results in a substantial loss of isometric force. We may conclude from these results that even during isometric contractions, sarcomere length non-uniformities develop naturally, they do not depend on active stretching on the descending limb of the force-length relationship, and they, in some unknown way, contribute to increased isometric force.

### Theoretical force-length relationship of single sarcomeres

When it was first demonstrated that force enhancement could be observed in a single sarcomere preparation (Leonard et al., 2010), critics were quick to point out the possibility that force enhancement could have been caused by the development of half-sarcomere non-uniformities. Although this argument sounds appealing on the surface, its acceptance leads quickly to a variety of unlikely consequences. Only one of these shall be discussed here.

**Figure 6:**
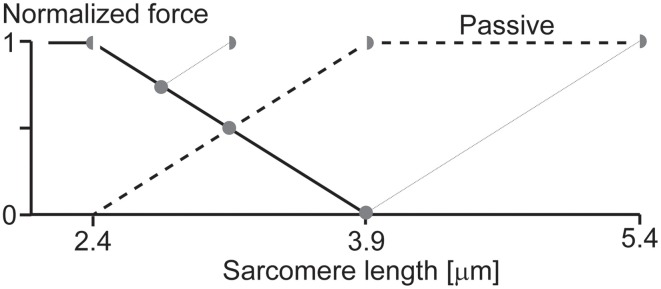
**Theoretical force-length relationship of a single sarcomere**. If we assume that force enhancement in a single sarcomere, as observed in the literature (Leonard et al., 2010), is caused by half-sarcomere length non-uniformities, and that active force is proportional to actin-myosin filament overlap, then the passive force would have to look as indicated in the figure (thick black dashed line). Such a passive force has never been observed and seems to contradict anything known about passive forces in skeletal muscles. Therefore, the notion that substantial force enhancement in a single sarcomere can be explained with the development of half-sarcomere length non-uniformities seems far-fetched, not to say impossible. Full circles indicate an entire sarcomere on the descending limb of the force-length relationship, as expected during an isometric contraction with equal half sarcomeres. Half-circles indicate the corresponding half-sarcomeres expected after an active stretch in the force enhanced state of a single sarcomere. Black line = descending limb of the active force-length relationship. Dashed black line = passive force required for the overextended half-sarcomere to match the force of the other, active, half sarcomere.

Imagine we have a single sarcomere just at the edge of the plateau leading to the descending limb of the force-length relationship (Figure 6) and we now stretch this sarcomere. For the sake of argument, let's also assume that at this initial length, there is no passive force (or if there was, we could subtract it from the initial considerations without affecting the following argument). Imagine now that we stretch this sarcomere, so its total length is ¼down, then ½down, and then at the full length down on the descending limb of the force-length relationship. If we now assume that one half of the sarcomere remains at its initial short length, then the other half would have to elongate twice the stretch magnitude of the whole sarcomere (Figure 6), and, for force equilibrium's sake, its force has to be equal to that of the half sarcomere that remained at its initial length. For this to occur, the passive force would have to start at the beginning (left hand side) of the descending limb of the force-length relationship, it would have to be a straight line, and its slope would have to be the same as that of the descending limb, except with opposite sign (positive instead of negative). Then, when the half sarcomere loses actin-myosin filament overlap, the passive force would have to remain constant. Needless to say, such a passive force has never been observed, and even if it had, it still could not explain the results by Leonard et al. (2010), where forces in the enhanced state clearly exceeded the isometric plateau forces in a single sarcomere preparation. This example illustrates how one has to be careful when making a proposal, or stating a critique, without thinking through the associated implications.

### A-band shifts and force enhancement

**Figure 7:**
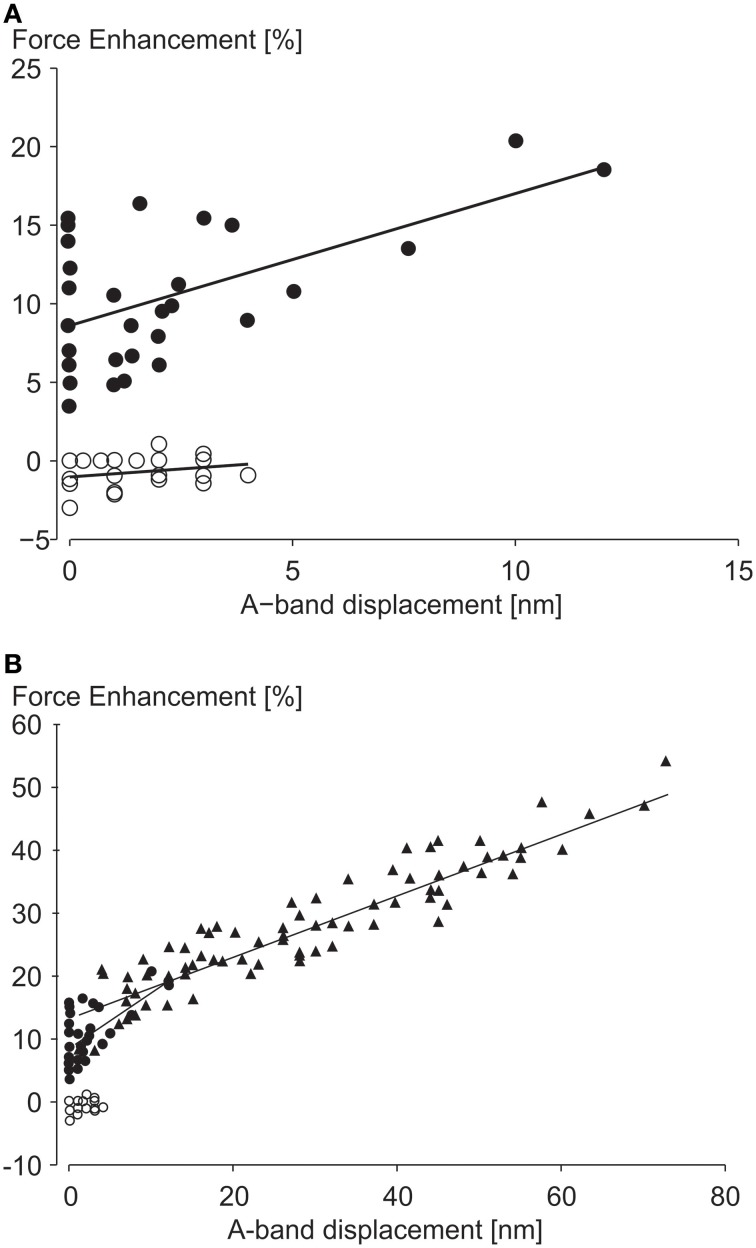
**Force enhancement as a function of half-sarcomere length non-uniformities**. **(A)** Force enhancement as a function of A-band displacements (equivalent to half sarcomere length non-uniformities) in single sarcomere preparations, and **(B)** in single myofibril preparations with multiple serial sarcomeres. Note that the small A-band shifts in the single sarcomere and the single myofibril only explain approximately 7 and 18% of the total force enhancement observed, assuming that the isometric reference contractions have perfectly identical half- and sarcomere length. However, since the isometric reference contractions have similar A-band shifts as the experimental active stretch contractions, none of the enhanced forces seems explained by stretch-induced half-sarcomere length non-uniformities from Rassier (2012) with permission.

In a recent study, A-band shifts, which indicate the degree of half-sarcomere length non-uniformities, were argued to “significantly increase the level of force enhancement (Rassier, 2012).” The argument was based on observed correlations of “stretch-induced” A-band shifts with residual force enhancement in single myofibril preparations (Figure 7). Here, a maximal A-band shift of approximately 72 nm was associated with an enhanced force of about 55%. However, a 72 nm shift would explain a 10% increase in force at best, thus most of the enhanced force remains unexplained. In a similar figure on single sarcomere experiments, with an implied spatial resolution of less than 0.5 nm, force enhancement for a 12 nm A-band shift was approximately 20%. A 12 nm shift explains a 1.5% increase in force leaving 92.5% of the observed force enhancement unexplained.

Moreover, experiments with no A-band shifts were found to have peak force enhancements of up to 16% (Figure 7). Finally, and probably most telling, half-sarcomere length non-uniformities for the isometric reference contractions, although not systematically evaluated in this study, were greater (their Figure 5C) or equal (their Figure 6C) to the non-uniformities of the actively stretched experimental contractions, thus it is hard to support the authors' claim that the force enhancement was associated with “stretch-induced” non-uniformities of half sarcomere lengths. Albeit not systematically evaluated, in studies where half-sarcomere length non-uniformities were compared for isometric reference and experimental stretch contractions, half-sarcomere lengths tended to be more uniform after stretch compared to reference contractions (Joumaa et al., 2007). Combined, these results provide little support that stretch-induced half-sarcomere non-uniformities contribute to the residual force enhancement in skeletal muscles.

## Conclusions and future directions

Ever since the emergence of the cross-bridge theory, properties of actively stretched muscles could not be predicted properly (Huxley, 1957). Forces and energy consumption were much too big compared to experimental results (Huxley, 1957), and residual force enhancement could not be predicted conceptually, as steady-state forces in the cross-bridge theory are independent of the history of contraction (Huxley, 1957; Huxley and Simmons, 1971; Walcott and Herzog, 2008). This shortcoming of the cross-bridge theory had been addressed by assuming that muscle segments (Hill, 1953) and sarcomeres (Morgan, 1990, 1994) were unstable on the descending limb of the force-length relationship, and small perturbations would cause great non-uniformities in sarcomere lengths. Active lengthening of muscles (eccentric contraction) was thought to be such a perturbation. It is interesting to note that the sarcomere length instability and associated stretch-induced development of sarcomere length non-uniformity theory has survived for such a long time, and in many circles is still unquestionably accepted, despite lack of direct evidence, and despite experimental results from the very beginning that were not in agreement with the predictions of the theory. For example, Abbott and Aubert (1952) had strong evidence of force enhancement on the ascending and plateau regions of the force-length relationship more than half a century ago, predating the formulation of the cross-bridge theory itself.

The refinement of mechanical experiments on single myofibrils (Bartoo et al., 1993; Rassier et al., 2003a; Joumaa et al., 2008; Leonard et al., 2010; Leonard and Herzog, 2010) and mechanically isolated sarcomeres (Leonard et al., 2010) has allowed for direct testing of many of the predictions of the sarcomere length non-uniformity theory. Among these rejected predictions (see discussion above), the ones most damaging to the non-uniformity thinking were the following:

1. The evidence that sarcomeres of vastly different length could reside side by side on the descending limb of the force-length relationship without appreciable length changes over periods of 30 s, thereby demonstrating stability;
2. That substantial force enhancement could occur in a single, mechanically isolated sarcomere;
3. And that the enhanced forces in single sarcomeres could exceed the purely isometric reference forces obtained at optimal sarcomere length by a substantial amount.

There is no doubt that sarcomeres in the force enhanced state are non-uniform. However, sarcomeres in muscles (Llewellyn et al., 2008), fibers (Huxley and Peachey, 1961) and myofibrils (Rassier et al., 2003a; Joumaa et al., 2008; Leonard and Herzog, 2010; Leonard et al., 2010) are also non-uniform for purely isometric contractions. Whether, or not these sarcomere length-non-uniformities increase with stretching has not been systematically elucidated, but pilot results, and isolated findings from unrelated studies, suggest that, if anything at all, sarcomeres following active muscle stretching are more stable (Edman et al., 1982) and have equal or less sarcomere length-non-uniformity compared to the corresponding isometric reference contractions (Joumaa et al., 2008; Rassier, 2012).

From these results, and evidence in the literature, we propose that sarcomere length non-uniformities are a normal associate of muscle contraction on all structural levels. They are not an occurrence exclusive to active muscle stretching (eccentric contractions) on the descending limb of the force-length relationship, and they are not a primary cause for the enhanced forces observed in skeletal muscles after active stretching.

**Figure 8:**
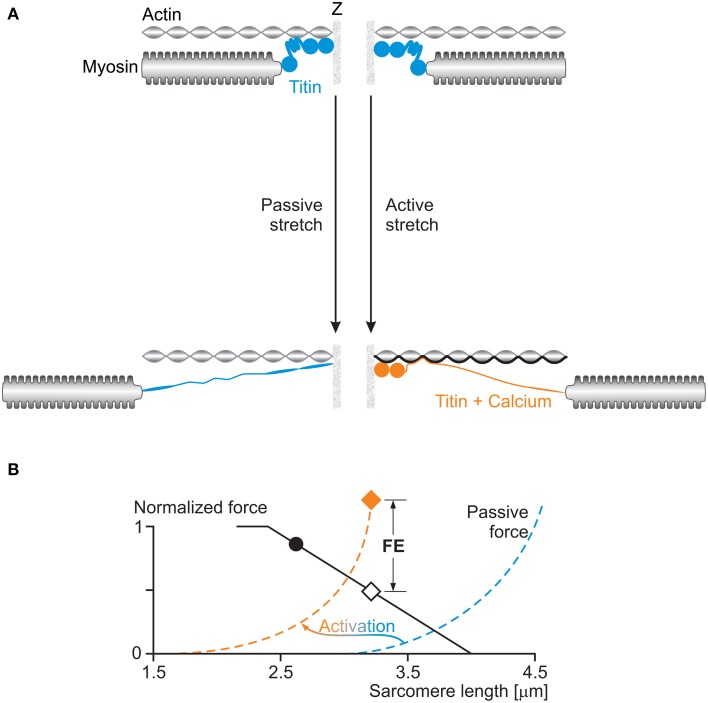
**(A)** Titin-induced force enhancement: a half-sarcomere is stretched passively (left) and actively (right). During passive stretching, titin elongates according to its normal spring properties. During active stretching, calcium binds to titin and titin's proximal region binds to actin: both of these events increase titin's stiffness and thus its force when actively stretched compared to when passively stretched. **(B)** The effects of activation on titin's force (passive force) are illustrated schematically in the force-length graph with a shift of the passive force to the left of the sarcomere length scale, and an increase in stiffness at a given sarcomere length.

If not the development of stretch-induced sarcomere length non-uniformities, how else can we explain residual force enhancement? A little over a decade ago, we discovered the existence of a passive component of residual force enhancement (Herzog and Leonard, 2002). Evidence accumulated over the past decade strongly suggests that the structural protein titin causes this passive force enhancement (Herzog et al., 2006). The idea was that titin, which acts as a molecular spring in the I-band region of sarcomeres, alters its spring stiffness when a muscle is activated. In principle, titin's stiffness can be changed in two ways: (i) by changing its material properties, or (ii) by changing its free spring length. Recent findings support the idea that both these mechanisms are at work in actively stretched skeletal muscles (Figure 8).

Titin is known to increase its stiffness upon muscle activation by binding calcium to specialized sites (Labeit et al., 2003; Joumaa et al., 2007). Labeit et al. (2003) identified the glutamate rich-region of the so-called PEVK segment of titin as a binding site for calcium, and Duvall et al. (2012) showed that calcium binds to specific Immunoglobulin (Ig) domains of titin and by doing so makes titin stiffer. There might be other calcium binding sites on titin that have yet to be identified.

Titin is also thought to change its free spring length by binding its proximal region to actin upon activation and muscle force production. Leonard and Herzog (2010) demonstrated that actively stretched sarcomeres and myofibrils produced about 3–4 times greater forces than passively stretched myofibrils in regions where actin-myosin filament overlap had been lost, and thus active forces were zero. Similarly, early findings on labeled titin segments in myofibrils indicate that during passive stretching, all I-band segments of titin elongate, as expected, while during active stretching, some proximal segments in the I-band region do not elongate, suggesting that they may be bound to a rigid backbone, for example the actin filament. In *mdm* knockout mice, where part of the N2A region of titin, thought to be critical for attachment of titin to actin, is eliminated, the difference between passive forces in actively and passively stretched myofibrils is small (about 15%) and can readily be explained with calcium binding to the titin segments identified above.

These results point to titin as a force regulating protein in muscle contraction; specifically in eccentric contractions and in the force enhanced state. Corresponding conceptual models of such a three filament force regulating sarcomere (Figure 8) have been discussed elsewhere in detail (Herzog et al., 2012a,b; Herzog, 2014) and a corresponding mathematical model has been developed and recently published (Schappacher-Tilp et al., 2015).

Independent of the ultimate explanation for residual force enhancement in skeletal muscles, the proposed substitution of the two filament (actin and myosin) with a three filament (actin, myosin, and titin) sarcomere model for force production has a variety of advantages over the sarcomere length non-uniformity theory, not the least of which is that it can explain all isometric and concentric force properties of the traditional cross-bridge model, but can also predict the increased force during eccentric contractions, the efficiency of eccentric contractions and the active and passive force enhancement following active muscle stretching.

But not only that, the three filament model of muscle force production has some intuitively appealing properties, including:

- The passive structures of muscles are soft and compliant when passively stretched but become hard and stiff during active stretching, thus providing additional force at negligible energetic cost.
- Titin forces increase when actin-myosin forces decrease, thereby providing a mechanism preventing damage in muscles stretched actively to long lengths.
- Titin provides stability for sarcomeres on the descending limb of the force-length relationship and for myosin filaments in the middle of the sarcomere. When titin is eliminated, all passive, and active force transmission across sarcomeres is lost (Leonard and Herzog, 2010).

### Conflict of interest statement

The authors declare that the research was conducted in the absence of any commercial or financial relationships that could be construed as a potential conflict of interest.
